# Population-based targeted sequencing of 54 candidate genes identifies *PALB2* as a susceptibility gene for high-grade serous ovarian cancer

**DOI:** 10.1136/jmedgenet-2019-106739

**Published:** 2020-06-16

**Authors:** Honglin Song, Ed M Dicks, Jonathan Tyrer, Maria Intermaggio, Georgia Chenevix-Trench, David D Bowtell, Nadia Traficante, AOCS Group, James Brenton, Teodora Goranova, Karen Hosking, Anna Piskorz, Elke van Oudenhove, Jen Doherty, Holly R Harris, Mary Anne Rossing, Matthias Duerst, Thilo Dork, Natalia V Bogdanova, Francesmary Modugno, Kirsten Moysich, Kunle Odunsi, Roberta Ness, Beth Y Karlan, Jenny Lester, Allan Jensen, Susanne Krüger Kjaer, Estrid Høgdall, Ian G Campbell, Conxi Lázaro, Miguel Angel Pujara, Julie Cunningham, Robert Vierkant, Stacey J Winham, Michelle Hildebrandt, Chad Huff, Donghui Li, Xifeng Wu, Yao Yu, Jennifer B Permuth, Douglas A Levine, Joellen M Schildkraut, Marjorie J Riggan, Andrew Berchuck, Penelope M Webb, OPAL Study Group, Cezary Cybulski, Jacek Gronwald, Anna Jakubowska, Jan Lubinski, Jennifer Alsop, Patricia Harrington, Isaac Chan, Usha Menon, Celeste L Pearce, Anna H Wu, Anna de Fazio, Catherine J Kennedy, Ellen Goode, Susan Ramus, Simon Gayther, Paul Pharoah

**Affiliations:** 1 Department of Oncology, University of Cambridge, Cambridge, Cambridgeshire, UK; 2 School of Women's and Children's Health, Faculty of Medicine, University of New South Wales, Sydney, New South Wales, Australia; 3 Cancer Genetics, Queensland Institute of Medical Research—QIMR, Herston, Queensland, Australia; 4 Cancer Genomics and Genetics and Women’s Cancer Programs, Peter MacCallum Cancer Centre, Melbourne, Victoria, Australia; 5 Sir Peter MacCallum Department of Oncology, The University of Melbourne, Melbourne, Victoria, Australia; 6 QIMR Berghofer Department of Genetics and Computational Biology, Herston, Queensland, Australia; 7 Department of Research, Peter MacCallum Cancer Centre, Melbourne, Victoria, Australia; 8 Cancer Research UK Cambridge Institute, University of Cambridge, Cambridge, Cambridgeshire, UK; 9 Laura and Isaac Perlmutter Cancer Center, New York University, New York, New York, USA; 10 Huntsman Institute, University of Utah, Salt Lake City, Utah, USA; 11 Division of Public Health Sciences, Fred Hutchinson Cancer Research Center, Seattle, Washington, USA; 12 Department of Epidemiology, University of Washington, Seattle, Washington, USA; 13 Department of Gynaecology, Jena University Hospital, Friedrich Schiller University Jena, Jena, Thüringen, Germany; 14 Gynaecology Research Unit, Hannover Medical School, Hannover, Niedersachsen, Germany; 15 Department of Radiation Oncology, Hannover Medical School, Hannover, Niedersachsen, Germany; 16 Department of Gynaecology, NN Alexandrov National Cancer Centre, Minsk, Minsk, Belarus; 17 Womens Cancer Research Center, Magee-Womens Research Institute, Pittsburgh, Pennsylvania, USA; 18 Department of Obstetrics, Gynecology and Reproductive Sciences, University of Pittsburgh School of Medicine, Pittsburgh, Pennsylvania, USA; 19 Division of Cancer Prevention and Control, Roswell Park Cancer Institute, Buffalo, New York, USA; 20 Department of Gynecologic Oncology, Roswell Park Cancer Institute, Buffalo, New York, USA; 21 School of Public Health, University of Texas Health Science Center at Houston, Houston, Texas, USA; 22 Department of Obstetrics and Gynecology, David Geffen School of Medicine, University of California Los Angeles, Los Angeles, California, USA; 23 Women's Cancer Program at the Samuel Oschin Comprehensive Cancer Institute, Cedars-Sinai Medical Center, Los Angeles, California, USA; 24 Department of Virus, Lifestyle and Genes, Danish Cancer Society Research Center, Kobenhavn, Denmark; 25 Department of Gynaecology, Rigshospitalet, University of Copenhagen, Kobenhavn, Denmark; 26 Department of Pathology, Herlev Hospital, University of Copenhagen, Kobenhavn, Denmark; 27 Department of Research, Cancer Genomics and Genetics, Peter MacCallum Cancer Centre, Melbourne, Victoria, Australia; 28 Hereditary Cancer Program, Catalan Institute of Oncology, Barcelona, Catalunya, Spain; 29 Translational Research Laboratory, Catalan Institute of Oncology, Barcelona, Catalunya, Spain; 30 Department of Laboratory Medicine and Pathology, Mayo Clinic, Rochester, Minnesota, USA; 31 Department of Health Science Research, Division of Biomedical Statistics and Informatics, Mayo Clinic, Rochester, Minnesota, USA; 32 Department of Epidemiology, University of Texas MD Anderson Cancer Center, Houston, Texas, USA; 33 Department of Cancer Epidemiology, Moffitt Cancer Center, Tampa, Florida, USA; 34 Department of Surgery, Memorial Sloan Kettering Cancer Center, New York, New York, USA; 35 Gynecologic Oncology, Laura and Isaac Pearlmutter Cancer Center, New York University, New York, New York, USA; 36 Department of Public Health Sciences, University of Virginia, Charlottesville, Virginia, USA; 37 Department of Gynecologic Oncology, Duke University Hospital, Durham, North Carolina, USA; 38 Population Health Department, QIMR Berghofer Medical Research Institute, Herston, Queensland, Australia; 39 Department of Genetics and Pathology, Pomeranian Medical University in Szczecin, Szczecin, Zachodniopomorskie, Poland; 40 Independent Laboratory of Molecular Biology and Genetic Diagnostics, Pomeranian Medical University in Szczecin, Szczecin, Zachodniopomorskie, Poland; 41 MRC Clinical Trials Unit, Institute of Clinical Trials & Methodology, University College London, London, London, UK; 42 Department of Epidemiology, University of Michigan School of Public Health, Ann Arbor, Michigan, USA; 43 Department of Preventive Medicine, Keck School of Medicine, University of Southern California, Los Angeles, California, USA; 44 Centre for Cancer Research, The Westmead Institute for Medical Research, The University of Sydney, Sydney, New South Wales, Australia; 45 Department of Gynaecological Oncology, Westmead Hospital, Westmead, New South Wales, Australia; 46 Department of Health Science Research, Division of Epidemiology, Mayo Clinic, Rochester, Minnesota, USA; 47 Kinghorn Cancer Centre, Garvan Institute of Medical Research, Darlinghurst, New South Wales, Australia; 48 Center for Bioinformatics and Functional Genomics and the Cedars Sinai Genomics Core, Cedars-Sinai Medical Center, Los Angeles, California, USA; 49 Department of Public Health and Primary Care, University of Cambridge, Cambridge, UK

**Keywords:** cancer: endocrine, genetic epidemiology

## Abstract

**Purpose:**

The known epithelial ovarian cancer (EOC) susceptibility genes account for less than 50% of the heritable risk of ovarian cancer suggesting that other susceptibility genes exist. The aim of this study was to evaluate the contribution to ovarian cancer susceptibility of rare deleterious germline variants in a set of candidate genes.

**Methods:**

We sequenced the coding region of 54 candidate genes in 6385 invasive EOC cases and 6115 controls of broad European ancestry. Genes with an increased frequency of putative deleterious variants in cases versus controls were further examined in an independent set of 14 135 EOC cases and 28 655 controls from the Ovarian Cancer Association Consortium and the UK Biobank. For each gene, we estimated the EOC risks and evaluated associations between germline variant status and clinical characteristics.

**Results:**

The ORs associated for high-grade serous ovarian cancer were 3.01 for *PALB2* (95% CI 1.59 to 5.68; p=0.00068), 1.99 for *POLK* (95% CI 1.15 to 3.43; p=0.014) and 4.07 for *SLX4* (95% CI 1.34 to 12.4; p=0.013). Deleterious mutations in *FBXO10* were associated with a reduced risk of disease (OR 0.27, 95% CI 0.07 to 1.00, p=0.049). However, based on the Bayes false discovery probability, only the association for *PALB2* in high-grade serous ovarian cancer is likely to represent a true positive.

**Conclusions:**

We have found strong evidence that carriers of *PALB2* deleterious mutations are at increased risk of high-grade serous ovarian cancer. Whether the magnitude of risk is sufficiently high to warrant the inclusion of *PALB2* in cancer gene panels for ovarian cancer risk testing is unclear; much larger sample sizes will be needed to provide sufficiently precise estimates for clinical counselling.

## Introduction

Rare, predicted deleterious variants in multiple genes have been shown to be associated with a moderate to high risk of epithelial ovarian cancer (EOC). These include the DNA double stand break repair genes *BRCA1*,^1^ *BRCA*,^2^ *BRIP1*,^3^ *RAD51C*, and *RAD51* 4, and the mismatch repair genes *MSH2* and *MSH6*.^5 6^ *ANKRD11, FANCM, PALB2* and *POLE* have recently been reported as possible susceptibility genes.^7–9^ Multiple common variants conferring weaker risk effects have also been identified,^10–17^ some of which modify EOC risk in carriers of more highly penetrant gene mutations.^18 19^

EOC is heterogeneous with five main histotypes: high-grade serous (HGSOC), low-grade serous, endometrioid, clear cell and mucinous ovarian cancer. These have different clinical characteristics and outcomes and are characterised by different germline and somatic genetic changes that result in the perturbation of different molecular pathways. For example, germline mutations in DNA double break repair genes predispose to HGSOC while germline mutations in mismatch repair genes increase risk of the endometrioid and clear cell histotypes.^6^

The known susceptibility alleles account for less than 50% of the excess familial risk of ovarian cancer, suggesting that other susceptibility genes and alleles exist.^15^ The unexplained genetic component of risk is likely to be made up of a combination of common genetic variants conferring weak effects and uncommon alleles conferring weak to moderate relative risks (less than 10-fold).

The aim of this study was to identify additional ovarian cancer susceptibility genes using case-control sequencing of candidate genes identified through various approaches including their known function in pathways that are associated with ovarian cancer development and from whole exome sequencing studies (WES) of ovarian cancer cases that have identified putative deleterious mutations in genes not previously evaluated for EOC risk.

## Material and methods

### Selection of candidate genes

#### Genes based on known biological function

As several EOC susceptibility genes are involved in DNA double-strand break repair and Fanconi anaemia (FA),^8^ we selected genes involved in these pathways. FA is a rare genetic disease characterised by chromosomal instability, hypersensitivity to DNA crosslinking agents, defective DNA repair, severe bone marrow failure, cancer susceptibility and many congenital defects. To date, 22 FA genes have been identified, of which eight have previously been evaluated in ovarian cancer case-control studies:^3 4 6 8^ *FANCD1*, *FNACJ (BRIP1)*, *FANCL*, *FANCN (PALB2)*, *FANCM*, *FANCO (RAD51C)*, *FANCS (BRCA1) and FANCV (MAD2L2*). We selected nine FA genes not previously studied in ovarian cancer: *FANCA*, *FANCB*, *FANCC*, *FANCD2*, *FANCE*, *FANCG*, *FANCI*, *FANCP (SLX4)*, *FANCW*. We also included *FANCN (PALB2*), which has been studied previously in ovarian cancer^3 9 20–22^ but its association with EOC risk is equivocal. Eight candidate genes involved in other aspects of DNA repair were also included: *ALKBH3*, *CHEK2*, *GTF2H4*, *POLE*, *POLK*, *RDM1* and *XRCC1*.

#### Genes from whole exome sequencing studies (WES)

Twelve genes (*BUB1B*, *C5orf28*, *C6*, *DNAJB4*, *EXO1*, *LIG4*, *MKNK2*, *MMRN1*, *PARP1*, *RAD52*, *SMC1A* and *SNRNP200*) were selected from WES analysis of EOC cases where putative deleterious (truncating) mutations were identified at a greater frequency in cases compared with publicly available WES data from controls reported by the NHLBI GO Exome Sequencing Project and The Exome Aggregation Consortium databases (http://exac.broadinstitute.org). Germline WES data for EOC cases were available for 412 HGSOC cases from the Cancer Genome Atlas ovarian cancer study; 513 ovarian cancer cases from an Australian case series 6; 97 familial non-*BRCA1*/*BRCA2* ovarian cancer cases from Gilda Radner Familial Ovarian Cancer Registry and 54 ovarian cancer cases from the UK Familial Ovarian Cancer Registry.

Four genes from these WES studies (*GANC*, *KNTC1*, *PSG6* and *UPK2*) were selected because more than one family member diagnosed with ovarian cancer from 10 familial cases carried the same truncating mutation in one of these genes.

Finally, 21 genes were selected from analyses of several other unpublished EOC WES studies (personal communications) where the frequency of truncating mutations was greater in cases compared with controls. These genes were *ANAPC2*, *CNKSR1*, *DUOX1*, *FBXO10*, *NAT10*, *OSGIN1*, *PAK4*, *PHF20L1*, *PIK3C2G*, *PTGER3*, *PTX3*, *RAD54B*, *RECQL, RIPK3, RNASEL*, *SMG5*, *SPHK1*, *SULT1C2*, *UHRF2*, *WNT5A* and *ZFHX3*.

### Study subjects

We used case-control data from targeted sequencing, exome and array-based genotyping.

#### Targeted sequencing

We included 5914 EOC cases and 5479 controls of European ancestries from 19 studies—13 case-control studies, 1 familial ovarian cancer study from Poland, 2 clinical trials and 3 case-only studies (online supplementary table 1).^14^ HGSOC cases were preferentially plated out for sequencing where possible.

10.1136/jmedgenet-2019-106739.supp1 Supplementary data

#### Exome sequencing

We extracted data on the 54 candidate genes from 829 case and 913 controls from two ovarian cancer case-control studies (MDA^23–25^ and NCO^14^) for which whole exome sequence data were available (online supplementary table 1).

#### Variants from genotyping array data

For genes that reached nominal significance in the combined analysis of the targeted sequencing and exome sequencing data, we extracted genotypes of any deleterious variants included on the OncoArray and UK Biobank Axiom Array. These two arrays were used to genotype up to 18 936 controls and 13 288 cases from the Ovarian Cancer Association Consortium (OCAC),^15^ 9725 controls and 858 cases from UK Biobank GWAS (https://www.ukbiobank.ac.uk/), respectively. Samples overlapped with the sequencing studies were excluded from the analysis.

All studies had ethics committee approval, and all participants provided informed consent.

### Sequencing methods

Target sequence enrichment followed by sequencing was performed on the coding sequence and splice-sites of *ALKBH3, ANAPC2, BUB1B, C5ORF28, C6, CHEK2, CNKSR1, DNAJB4, DUOX1, EXO1, FANCA, FANCB, FANCC, FANCD2, FANCE, FANCG, FANCI, FBXO10, GANC, GTF2H4, KNTC1, LIG4, MKNK2, MMRN1, NAT10, OSGIN1, PAK4, PALB2, PARP1, PHF20L1, PIK3C2G, POLE, POLK, PSG6, PTGER3, PTX3, RAD52, RAD54B, RDM1, RECQL, REV3L, RIPK3, RNASEL, SLX4, SMC1A, SMG5, SNRNP200, SPHK1, SULT1C2, UHRF2, UPK2, WNT5A, XRCC1 and ZFHX3*. The target sequence was identified from the NCBI Reference Sequence Database48.48 Fluidigm access arrays as previously described.^6^ A total of 1663 amplicons were designed to cover the 159 kb target region. Libraries were sequenced using 150 bp paired-end sequencing on the Illumina HiSeq4000 or HiSeq2500.

Sequencing reads were demultiplexed and then aligned against the human genome reference sequence (hg19) using the Burrows-Wheeler Aligner.^26^ The Genome Analysis Toolkit^27^ was used for base quality-score recalibration, local indel realignment and variant calling. Finally, ANNOVAR^28^ was used for variant annotation. Variants were called if (1) genotype information was available from a chip genotype for that sample or (2) the variants were presented in more than one amplicon or (3) read depth ≥15 and alternate allele frequency ≥40% or (4) read depth ≥100 and alternate allele frequency ≥25%. These thresholds were defined using the results from sequencing of positive controls with known variants and genotype information from chip array genotyping of overlapping samples.

We excluded 356 cases and 269 controls because <80% of the target sequence bases had a read depth of at least 15. The average percentage coverage of the genes at 15X read depth ranged from 64% to 99% (online supplementary table 2). The mean sequencing depth for these genes ranged from 130 (IQR 104–152) to 432 (IQR 364–492). Concordance for 111 duplicate pairs was 98% (7384 concordant variants out of total 7572 variants called).

For the exome sequencing, sonication fragmentation was used to fragment DNA samples. Fragments with an average size of 200 bp were selected to generate libraries for sequencing. Agilent SureSelect Clinical Research Exome (CRE) v1 was used for exome enrichment and sequencing was performed on an Illumina HiSeq 4000 using 2×150 bp paired-end reads. Cutadapt (https://doi.org/10.14806/ej.17.1.200) was used to locate and remove residual adapters in reads. FLASH (Fast Length Adjustment of SHort reads)^29^ was used to merge the overlapped paired-end reads into one read, using default parameters. Reference genome alignment and joint genotype calling according to a pipeline described in Yu *et al*.^30^ The coding sequences and splice sites of all 54 genes were extracted. Fifty-three genes with 100% average coverage at 10X were included in the analysis. *GTF2H4* was excluded from the analysis, as the average coverage was only 43%.

Deleterious variants were defined as those predicted to result in protein truncation (frameshift indel, splice site, nonsense mutations and start loss) or predicted to be deleterious and/or likely deleterious by Clinvar.^31^ Any exonic single nucleotide variants within 3 bp of the exon-intron boundary and any intronic variants within 20 bp of the exon-intron boundary at the 5-prime end, and 6 bp at the 3-prime end, were evaluated using the software MaxEntScan to identify those most likely to disrupt splicing.^32^ Variants with a MaxEntScan score that decreased by more than 40% compared with the reference sequence and having a reference sequence score ≥3 were considered deleterious. Sequencing alignments were confirmed by visual inspection using the Integrative Genomic Viewer.^33^

### Statistical methods

#### Risk estimation and genotype-phenotype analyses

We used a simple burden test for association between deleterious variants and ovarian cancer risk on a gene-by-gene basis. The burden test was based on unconditional logistic regression adjusted for country (Australia, Denmark, German, Poland, the UK and the USA) and sequencing method (targeted sequencing or exome sequencing). ORs and associated 95% CI were calculated.

#### Missense variant analyses

We also identified multiple rare (minor allele frequency <1%) missense variants that have an unknown functional effect on the protein. We used the rare admixture likelihood burden test^34^ to test these variants for association. We excluded any missense variants classified as deleterious and classified the remaining variants by whether or not they are predicted to have a damaging effect on protein function by two out of three prediction tools—SIFT (score <0.05),^35^ polyphen-2^36^ (classified as probably damaging or damaging) and Provean^37^ (score≤−2.5). Subjects with a missense variant call rate less than 80% and variants with a call rate less than 80% or with genotype frequencies inconsistent with Hardy-Weinberg equilibrium (p<10^–5^) were excluded.

## Results

### Germline deleterious mutations in ovarian cancer cases and controls

Sequencing results were available for 6385 EOC cases and 6115 controls after quality control analysis. The characteristics of these individuals by study are summarised in online supplementary table 1. Most EOC cases were serous histotype (n=6304, 98.7%), of which 5951 were the HGSOC histotype (93.2%).

We identified 629 unique, putative-deleterious variants (online supplementary table 3) in 1051 ovarian cancer cases (967 high-grade serous histotype) and 964 controls. There was a nominally significant higher frequency of mutations in cases compared with controls for *POLK*, *PALB2* and *SLX4* and a lower frequency of mutations in cases compared with controls for *FBXO10* (table 1). The associated ORs are shown in table 1—for *POLK, PALB2* and *SLX4* the effect size was slightly larger for HGSOC. The frequency of deleterious variants in the other genes was similar in cases compared with controls (online supplementary table 4). Given the evidence for association of multiple FA genes with EOC risk, we also carried out a burden test to compare the frequency of deleterious variants in any of the eight genes which were not significantly associated with ovarian cancer risks individually (*FANCA*, *FANCB*, *FANCC*, *FANCD2*, *FANCE*, *FANCG, FANCI* and *FANCL*). A combined analysis will have greater power if multiple genes were associated but the effect sizes too small to detect individually. There was no significant difference in the frequency of deleterious variants in cases (96/6184, 1.6%) and controls (85/6089, 1.4%) (p=0.50).

**Table 1:** Frequency of mutations and estimated risk of EOC in candidate genes (p<0.05) from targeted sequencing and exome sequencing

Set*	Histotype	Gene	Controls		Cases		Or (95% CI)	P value
			No.	%	No.	%		
TS	Overall	*POLK*	9	0.17	29	0.52	3.04 (1.43 to 6.43)	0.0037
		*PALB2*	6	0.12	19	0.34	3.10 (1.23 to 7.78)	0.016
		*SLX4*	4	0.08	13	0.23	3.08 (1.00 to 9.48)	0.0049
		*FBXO10*	9	0.17	3	0.053	0.30 (0.08 to 1.11)	0.071
		Non-carrier	5174	99.5	5492	98.8		
	HGSOC	*POLK*	9	0.17	27	0.53	3.17 (1.48 to 6.79)	0.003
		*PALB2*	6	0.12	18	0.35	3.30 (1.30 to 8.38)	0.012
		*SLX4*	4	0.08	13	0.25	3.51 (1.13 to 10.9)	0.029
		*FBXO10*	9	0.17	3	0.059	0.32 (0.09 to 1.18)	0.086
		Non-carrier	5174	99.5	5062	98.8		
ES	Overall	*POLK*	7	0.77	6	0.72	0.94 (0.32 to 2.82)	0.92
		*PALB2*	2	0.22	3	0.36	1.65 (0.28 to 9.93)	0.58
		*SLX4*	0	0	2	0.24	NA	
		*FBXO10*	1	0.11	0	0		
		Non-carrier	903	98.9	818	98.7		
	HGSOC	*POLK*	7	0.77	6	0.72	0.94 (0.32 to 2.82)	0.92
		*PALB2*	2	0.22	3	0.36	1.66 (0.28 to 9.94)	0.58
		*SLX4*	0	0	2	0.24	NA	
		*FBXO10*	1	0.11	0	0	NA	
		Non-carrier	903	98.9	817	98.7		

*TS: targeted sequencing; ES: exome sequencing.

EOC, epithelial ovarian cancer; HGSOC, high-grade serous ovarian cancer; OCAC, Ovarian Cancer Association Consortium.

### Validation analyses in ovarian cancer case-control studies

We also evaluated risk associations between deleterious variants in *POLK*, *PALB2*, and *SLX4* with EOC risk based on germline genotyping data for 13 277 EOC cases and 18 930 controls from OCAC and for 858 EOC cases and 9725 controls and from UK Biobank. For OCAC samples, data were available for six deleterious non-monomorphic variants in *PALB2*; for UK Biobank samples, data were available for seven *PALB2* and one *POLK* deleterious variants (table 2, list of variants in online supplementary table 5).

**Table 2:** Frequency of mutations and estimated risk of EOC in candidate genes for validation chip genotyping data

Set*	Histotype	Gene	Controls		Cases		OR (95% CI)	P value
			No.	%	No.	%		
OCAC	Overall	*PALB2*	6	0.03	11	0.08	2.10 (0.74 to 5.94)	0.16
		Non-carrier	18 930	99.97	13 277	99.9		
	HGSOC	*PALB2*	6	0.03	6	0.097	3.48 (1.10 to 11.1)	0.035
		Non-carrier	18 930	99.97	6168	99.9		
Biobank	Overall	*PALB2*	11	0.11	3	0.35	3.12 (0.87 to 11.2)	0.081
		*POLK*	29	0.30	2	0.23	0.78 (0.19 to 3.29)	0.74
		Non-carrier	9685	99.6	853	99.4		
	HGSOC†	*PALB2*	11	0.11	1	0.28	2.49 (0.32 to 19.4)	0.38
		*POLK*	29	0.30	1	0.28	0.92 (0.12 to 6.74)	0.93
		Non-carrier	9685	99.6	361	99.4		

*OCAC: OCAC sample genotype on the OncoArray; Biobank: genotype from UK Biobank Axiom Array.

†Information on tumour grade was not available for UK Biobank cases, all the serous cases in UK Biobank were assumed to be HGSOC.

EOC, epithelial ovarian cancer.

In OCAC case-control analyses, *PALB2* variants showed a non-significant increased risk of EOC (OR 2.10, 95% CI 0.74 to 5.94, p=0.16). The strength of this association increased when the analysis was restricted to 6181 HGSOC cases (OR 3.48, 95% CI 1.10 to 11.1, p=0.035). In UK Biobank, we observed a weak association for *PALB2* mutations with EOC risk (OR 3.12, 95% CI 0.87 to 11.2, p=0.081). There was no evidence of risk association for mutations in *POLK* (table 2).

We then performed a meta-analysis by combining the targeted sequencing, WES and chip genotyping data. Taken together, putative deleterious mutations were associated with increased risk for *PALB2* (OR 2.60, 95% CI 1.45 to 4.64; p=0.0013), *POLK* (OR 1.77, 95% CI 1.07 to 2.93; p=0.026) and *SLK4* (OR 3.37, 95% CI 1.17 to 9.70, p=0.024) and decreased risk for *FBXO10* (95% CI 0.07 to 1.00; p=0.049). After stratifying cases by histological subtype, the estimated risks were higher for HGSOC for *PALB2* (OR 3.01, 95% CI 1.59 to 5.68; p=0.00068), *POLK* (OR 1.99, 95% CI 1.15 to 3.43; p=0.014) and *SLK4* (OR 3.92, 95% CI 1.33 to 11.5; p=0.013).

We used an approximate Bayes factor to calculate the Bayes false discovery probability (BFDP) described by Wakefield^38^ for *PALB2*, *SLX4*, *POLK* and *FBXO10* based on several different priors and assuming that the associated risk is unlikely to be greater than an OR of 4 (table 3). The evidence for association of *PALB2* was strong with a BFDP of less than 15% when the prior on the alternative hypothesis is 0.1. The nominally significant associations for the other three genes are likely to be false positives.

**Table 3:** Bayes false discovery probability for the associations reported for *PALB2, SLX4, POLK and FBXO10* from the meta-analysis of all the available data

Gene	Histotype	OR (95% CI)	P value	Prior probability		
				0.1	0.05	0.01
*PALB2*	HGSC	3.01 (1.59 to 5.68)	0.00068	0.14	0.26	0.65
*SLX4*	HGSC	3.92 (1.33 to 11.5)	0.013	0.65	0.80	0.95
*POLK*	HGSC	1.99 (1.15 to 3.43)	0.014	0.65	0.80	0.95
*FBXO10*	Overall	0.27 (0.07 to 1.00)	0.026	0.75	0.86	0.97

### Predicting the functional impact of missense coding variants

Combining the whole exome and targeted sequencing data, we identified 5265 unique missense variants with minor allele frequency less than 1% in the 54 genes (online supplementary table 6). We used the in silico software programs SIFT, Polyphen-2 and Provean to evaluate the predicted impact of these variants on protein function for each gene. Of the 5265 variants, 2111 were classified as ‘deleterious’ based on at least 2 out of 3 of these classifiers. We found weak evidence for association with increased EOC risk for rare missense variants in *DUOX1* and *PAK4* using burden testing (p=0.015 and 0.025, respectively) (online supplementary table 7); for *DUOX1*, the strength of this association improved when the analyses were restricted to the HGSOC histotype (p=0.0061). When we performed the same analyses for 1493 very rare variants (MAF<0.001), we observed significant association for missense variants in *DUOX1* and *FANCE* (p=0.015 and 0.034, respectively).

## Discussion

We have evaluated the association between putative deleterious variants in 54 genes with the risk of HGSOC through a combination of whole exome and targeted sequencing analysis in 5951 cases and 6115 controls of broad European ancestries. We found evidence for four genes—*PALB2*, *POLK*, *SLX4* and *FBXO10*—associated with HGSOC risk. Association analysis in an additional 14 135 ovarian cancer cases and 28 655 controls genotyped through OCAC and the UK Biobank provided further support for *PALB2* as a HGSOC susceptibility gene.

The probability that a genetic association deemed statistically significant is a false positive depends on the prior of the null hypothesis and the power of the study to detect an effect size plausible under the alternative hypothesis. We calculated Wakefield’s BFDP^38^ based on several different priors to further evaluate the likelihood that *PALB2*, *POLK, SLX4* and *FBXO10* are EOC susceptibility genes. If we assume the prior on the alternative to be 1 in 10 or 1 in 20, the BFDPs for the association of deleterious variants in *PALB2* with HGSOC are 0.14 and 0.26, respectively. These moderately strong priors are reasonable given the evidence for the association from previously published studies.^20^ Two studies have reported nominally significant associations for *PALB2* with OR 4.4 (95% CI 2.1 to 9.1)^20^ and (2.87, 95% CI 1.61 to 4.74).^21^ Kotsopoulos and colleagues reported an increased risk that was not significant (OR, 4.55, 95% CI 0.76 to 27) and, in a subset of the samples included in this study, we also found a non-significant increase in risk (OR 3.2, 95% CI 0.86 to 12).^3^

It is possible that cryptic population structure could cause spurious association in these data. Principal component analysis is one approach to reducing the risk of such bias, but there are too few common variants in the regions covered by the targeted sequencing panel to do a principal component analysis and chip genotyping data that would be required for such an analysis is not available for all the samples. Adjusting for country of origin and restricting the analysis to samples from individuals of broad European ancestries should reduce any problem with population stratification.

**Figure 1:**
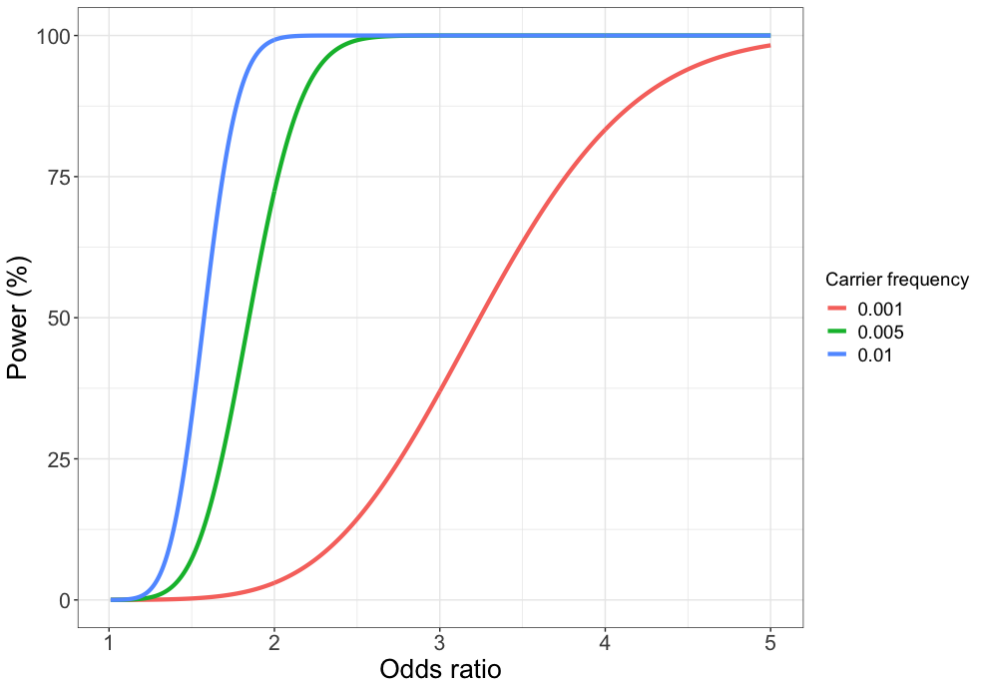
Power to detect association for 5951 cases and 6385 controls at a Type I error rate of 0.0001 by deleterious variant carrier frequency and effect size (OR).

We lacked the statistical power to identify susceptibility genes conferring relative risks of less than 2 (figure 1). Our use of targeted sequencing and a definition of deleterious variants as those that likely truncate the protein product will have probably underestimated the true prevalence of deleterious variants in these genes. Incomplete coverage of each gene will have missed some small indels and single nucleotide variants. Amplicon based sequencing will also miss large deletions and rearrangements, which are relatively common in some genes.^39 40^ Finally, any functional mutations in the non-coding region of these genes will have been missed.^41^

**Figure 2:**
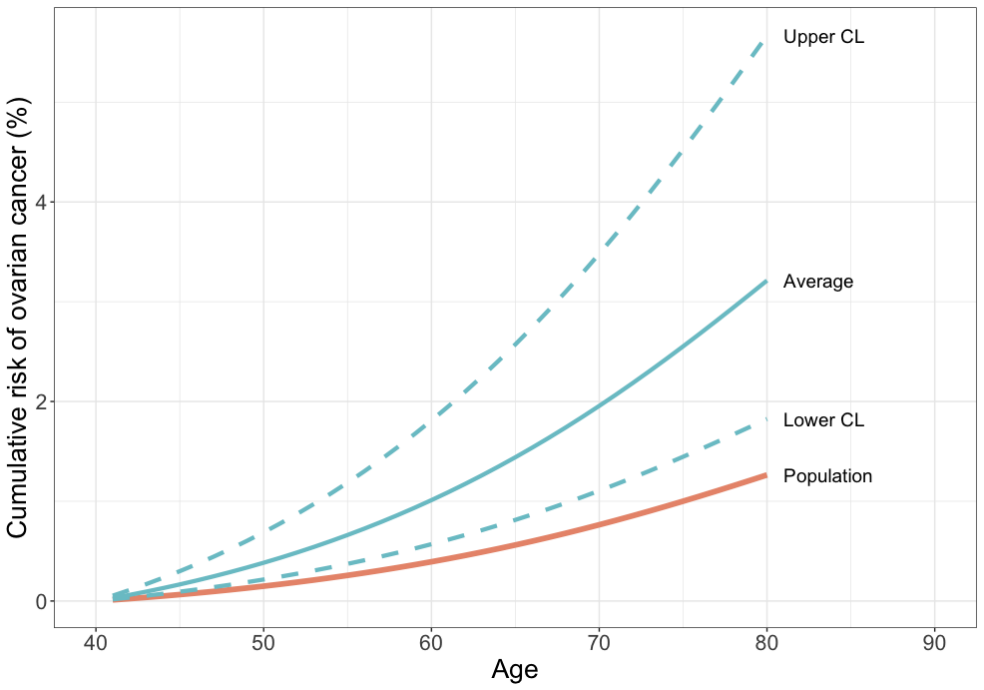
Estimated cumulative risk (%) of ovarian cancer in a *PALB2* deleterious variant carrier compared with population risks for England and Wales, 2016.

Some commercial gene-panel tests for hereditary breast-ovarian cancer already include *PALB2*. However, whether there is clinical utility in testing unaffected women for deleterious mutations in *PALB2* is not clear given the uncertainties in the risk estimates for this gene. There is no consensus over the risk threshold at which preventative surgery should be offered; many cancer genetics clinics in the UK will refer women if their predicted lifetime risk of EOC is greater than 10%. Others have suggested that the risk threshold should be lower given the low risk nature of the intervention; prophylactic surgery has been shown to be cost-effective for women at a lifetime risk of 5%. Recent updates to the US National Comprehensive Cancer Network Guidelines recommend considering risk reducing salpingo-oophorectomy in carriers of moderate risk genes if the lifetime risk of such mutation carriers exceeds 2.6%. Based on our data and population data for ovarian cancer incidence in England and Wales in 2016, the cumulative risk of ovarian cancer by age 80 for a carrier of a deleterious *PALB2* mutation is 3.2% (figure 2). Thus, a woman carrying a *PALB2* deleterious mutation would be eligible for prophylactic surgery. However, the CIs for this estimate range from 1.8% to 5.7%. Very large, well-designed case-control studies will be required to provide more precise, unbiased estimates of risk suitable for clinical counselling.

In summary, we have found relatively strong evidence that deleterious germline mutations in *PALB2* are associated with a moderate increase in the risk of HGSOC with weak evidence for *POLK*, *SLX4* and *FBXO10*. Mutations in the other 50 genes we tested are unlikely to contribute meaningfully to genetic predisposition to HGSOC. This study highlights the importance of large sample sizes needed to obtain risk estimates with the precision necessary for clinical use.

## Data Availability

Data are available from the authors on request.
